# Sulforaphane Bioavailability from Glucoraphanin-Rich Broccoli: Control by Active Endogenous Myrosinase

**DOI:** 10.1371/journal.pone.0140963

**Published:** 2015-11-02

**Authors:** Jed W. Fahey, W. David Holtzclaw, Scott L. Wehage, Kristina L. Wade, Katherine K. Stephenson, Paul Talalay

**Affiliations:** 1 Department of Pharmacology and Molecular Sciences, Johns Hopkins University School of Medicine, Baltimore, Maryland, United States of America; 2 Department of International Health, Johns Hopkins University Bloomberg School of Public Health, Baltimore, Maryland, United States of America; National Institutes of Health, UNITED STATES

## Abstract

Glucoraphanin from broccoli and its sprouts and seeds is a water soluble and relatively inert precursor of sulforaphane, the reactive isothiocyanate that potently inhibits neoplastic cellular processes and prevents a number of disease states. Sulforaphane is difficult to deliver in an enriched and stable form for purposes of direct human consumption. We have focused upon evaluating the bioavailability of sulforaphane, either by direct administration of glucoraphanin (a glucosinolate, or β-thioglucoside-*N*-hydroxysulfate), or by co-administering glucoraphanin and the enzyme myrosinase to catalyze its conversion to sulforaphane at economic, reproducible and sustainable yields. We show that following administration of glucoraphanin in a commercially prepared dietary supplement to a small number of human volunteers, the volunteers had equivalent output of sulforaphane metabolites in their urine to that which they produced when given an equimolar dose of glucoraphanin in a simple boiled and lyophilized extract of broccoli sprouts. Furthermore, when either broccoli sprouts or seeds are administered directly to subjects without prior extraction and consequent inactivation of endogenous myrosinase, regardless of the delivery matrix or dose, the sulforaphane in those preparations is 3- to 4-fold more bioavailable than sulforaphane from glucoraphanin delivered without active plant myrosinase. These data expand upon earlier reports of inter- and intra-individual variability, when glucoraphanin was delivered in either teas, juices, or gelatin capsules, and they confirm that a variety of delivery matrices may be equally suitable for glucoraphanin supplementation (e.g. fruit juices, water, or various types of capsules and tablets).

## Introduction

The effectiveness of high consumption of plant-based diets in reducing the risk of cancer and many other chronic diseases is widely recognized. Evidence for the special protective effects of cruciferous plants (broccoli, cauliflower, cabbage, etc.) is very strong and has been attributed largely to their high content of glucosinolates. Whereas glucosinolates are not active anticarcinogens, they are converted by both the plant enzyme myrosinase and by the microflora of the gastrointestinal tract [1] to isothiocyanates which are extremely effective blockers of carcinogenesis [2–4]. A widely studied example is the presence in broccoli (and especially in 3-day-old broccoli sprouts) [5–8] of the glucosinolate glucoraphanin (GR) which is converted by the endogenous enzyme, myrosinase, to the isothiocyanate sulforaphane (SF; a phytochemical known also as a mustard oil). Whereas its modes of action as a chemoprotective agent are manifold, upregulating the “phase 2 response” consisting of the repressor protein Kelch-like ECH-associated protein 1 (Keap1), nuclear factor erythroid 2 p45-related factor 2 (Nrf2), and genes which contain an antioxidant responsive element (ARE), (frequently referred to as the Keap1-Nrf2-ARE pathway), is the best-studied of the mechanisms by which sulforaphane appears to protect the body against a variety of chronic diseases. Sulforaphane is a highly reactive compound. In plants, it occurs as its relatively inert precursor, glucoraphanin, which behaves as a pro-drug. More than two decades after its rediscovery [9], SF remains one of the most potent known naturally occurring inducers of cytoprotective phase 2 enzymes [2]. Upon consumption of myrosinase-containing plants and subsequent rupture of cells (e.g. by herbivorous insects, fungal or bacterial pathogens, or chewing humans) glucoraphanin is hydrolyzed and myrosinase, normally segregated from its substrate, is released [10,11,12]. The rapid product of enzymatic breakdown is spontaneously converted to, among other metabolites, sulforaphane. Sulforaphane acts as an antifeedant (deterring insect predators), a selective antibiotic, and a cytotoxin at high concentrations that may be encountered at plant wound sites [12]. In humans, sulforaphane is metabolized by initial rapid and reversible conjugation with glutathione, and successive steps of hydrolysis of the conjugates leading to ultimate formation of the *N*-acetylcysteine derivatives (known as mercapturic acids). All of these conjugates are chemically designated dithiocarbamates (DTC) and can be quantified by the cyclocondensation reaction developed in our laboratory [13].

Two types of highly standardized, lyophilized boiling water extracts of 3-day-old broccoli sprouts (BSE) have been prepared at Johns Hopkins University Medical School (JHU), utilizing good manufacturing (GMP) conditions, and have been used widely under clinical protocols approved by IRBs and under INDs approved by the FDA. These preparations are: (i) BSE containing mostly glucoraphanin; (ii) BSE that has been treated with homogenates of daikon (*Raphanus sativus* or Japanese radish) which is an extremely rich source of myrosinase that hydrolyzes GR to SF. The latter preparations contain sulforaphane and only trace amounts of glucoraphanin. These preparations have been extensively characterized analytically with respect to their content of active ingredients and their safety for human consumption.

We and others have used broccoli sprout extracts (both freshly made, and lyophilized) as a delivery agent for sulforaphane in clinical studies conducted in China, the USA, and the United Kingdom [14–17]. The use of such extracts, though effective, is complicated by the fact that sulforaphane is only moderately stable over time, especially in aqueous solution. The reactivity of sulforaphane is exacerbated by the fact that lyophilized extracts are hygroscopic, and as water is adsorbed during protracted storage or formulation, their useful shelf-life is limited unless chemically stabilized, kept cold, or made frequently during the study. It is difficult and expensive to stabilize and formulate sulforaphane for extended clinical trials and particularly for long-term interventions. Our long-term interest in food-based approaches [8,18], and successful use of them in clinical studies [6,7], has underscored a unique series of logistic and measurement challenges for an intervention trial using either broccoli or broccoli sprouts.

Administration of glucoraphanin as an oral precursor of SF results in highly variable conversions of GR to SF metabolites among volunteers, ranging from 1–40% [1,7,14–16,19,20], whereas 70–90% of oral SF is consistently converted to urinary dithiocarbamate metabolites in all subjects who have been studied [7,19]. In brief, freeze-dried GR-rich BSE has a very long shelf life (is very stable), and when dosed orally to humans, conversion of GR to SF, and absorption and metabolism of the active principle is quite variable. On the other hand, SF-rich BSE must be kept frozen to maintain potency, but is highly bioavailable when ingested, and there is low inter- and intra-individual variability in percent of dose excreted as SF dithiocarbamate metabolites [7,19].

We have thus focused upon evaluating the bioavailability of GR, using delivery systems that permit it to be converted to sulforaphane at economic, reproducible, and sustainable yields. We compare the bioavailability of a simple powder made by lyophilizing a boiling water extract of glucoraphanin-rich broccoli sprouts that contains no active myrosinase, with equimolar quantities of a commercially produced extract (a “dietary supplement”) derived from broccoli seeds. We also compare it to two very different preparations containing active myrosinase (broccoli seed powder and freeze-dried broccoli sprouts). Multiple sample matrices and modes of delivery show that regardless of delivery method, providing active myrosinase as part of the preparation enhanced the bioavailability of sulforaphane and reduced the variability of conversion (glucoraphanin to sulforaphane) that could otherwise be ascribed solely to myrosinase activity of the gut microbiota. Mean bioavailability of a range of glucoraphanin-rich preparations lacking active myrosinase was roughly 10% of dose as we have shown in previous studies [1,7,13,19], whereas when active myrosinase was included in the dose, bioavailability increased to almost 40%. Both within-subject and between-subject variability was also reduced, and when hydrolysis was accomplished *ex-vivo* by a short incubation prior to dosing, bioavailability was approximately 90%, confirming that the hydrolysis was most likely mediated by myrosinase present in the dose, although intestinal microbial metabolism is expected to also contribute to GR to SF conversion.

## Materials and Methods

### Broccoli Preparations and Delivery

Broccoli sprout extracts (BSE) were prepared by Johns Hopkins University investigators as described previously [7,14,15,20]. Both sulforaphane (SF)- and glucoraphanin (GR)-rich broccoli sprout extracts were prepared. These extracts have been the subjects of numerous clinical trials both with- and without INDs.

Broccoli seed extracts (BSdE) are now available commercially. Brassica Protection Products LLC (Baltimore, MD, USA) produced the broccoli seed extract utilized in this study, which was donated and encapsulated by one of the companies that produces supplements containing this extract (Xymogen, Orlando, FL, USA). This group also encapsulated our GR-rich broccoli sprout extract in identical green opaque “0” gel-caps at comparable low- and high-dose levels (69 and 230 μmol of GR), equivalent to what are in their high-dose and low-dose retail “OncoPLEX™” capsules. In order to do this, inclusion of small amounts of inert excipients commonly used in the supplement and pharmaceutical industry (e.g. stearic acid, magnesium stearate, precipitated silica, and microcrystalline cellulose) were required.

Freeze dried broccoli sprouts (FDBS) were grown under contract at a commercial green-sprouting facility (Hanover Foods Corp. / Sunsprout, Ridgley, MD, USA), using seeds selected and provided by JHU. Fresh sprouts were delivered to Johns Hopkins University, and immediately quick-frozen, and lyophilized. Lyophilized sprouts were tested for microbial contaminants, comminuted to a powder, and either bulk-packaged or encapsulated in standard gel-caps (gelatin #30-3533-1000, PCCA, Houston, TX, USA) or acid-resistant gel-caps (DRcaps, Capsugel, Morristown, NJ, USA).

Broccoli seed powder (BSdP) was made by surface disinfesting seeds of broccoli (*Brassica oleracea* var. *italica*) with 25% Clorox bleach, followed by extensive rinsing with distilled water and drying. Dried seeds were ground to a powder in a manner that prevented excessive heat formation which could have denatured the enzyme myrosinase. This seed preparation was thus rich in glucoraphanin and active myrosinase (which is present in both seeds and sprouts), but contained no sulforaphane. Extensive microbiological testing was performed, as were analytical tests to ensure the absence of both chemical and biological contaminants (e.g. pathogens and heavy metals).

### Analyses of Preparations

The GR content of the broccoli sprout preparations used in this study was determined using paired-ion high performance liquid chromatography (HPLC) and hydrophilic interaction HPLC [21,22]. GR was quantitated by comparing peak areas with the peak areas of an authentic GR standard prepared as previously described [23].

The absence of SF in the GR-rich preparations utilized herein was determined by HPLC as previously described [24], supplemented by the cyclocondensation reaction [13,25], that is further described below.

Myrosinase (thioglucoside glucohydrolase, E.C. 3.2.1.147) activity was determined spectrophotometrically by measuring the rate of decrease in absorbance at 227 nm due to the hydrolysis of the glucosinolate sinigrin, as previously reported [10,11]. One unit of activity is equal to the hydrolysis of 1 μmole of sinigrin per min.

All preparations were sent to a commercial microbiological testing laboratory (IEH-JL Analytical, Modesto, CA, USA) for testing prior to being administered to volunteers.

### Clinical Study Design

All subjects were instructed to avoid consumption of cruciferous vegetables in any form, or any other crucifer-containing foods, for at least three days prior to the start of each visit and during the 24 hours after dosing. They were provided with a list of vegetables and condiments to avoid. The night before the study, subjects were required to fast from midnight until after ingesting the dose the next morning. On the days of the interventions (broccoli consumption) the subjects were to arrive at the study site at 8:00 AM. They provided a pre-dose urine sample for the purpose of determining baseline DTC levels. At approximately 8:30 AM they ingested one of the broccoli preparations described herein. They were each given a bottle of spring water to drink *ad libitum*. After ingestion of the dose they were required to collect all of their post-dose urine until 8:30 AM the following day (24 hr), and they were provided with labeled bottles for this purpose. The volumes of the 24-hr urine collections were measured, and aliquots of these and the baseline urines were saved and either assayed immediately for DTC determination or frozen at -20°C until assayed.

Doses were delivered in a variety of vehicles or matrices: BSE (about a gram or less, depending on dose level) was: (a) mixed rapidly in room temperature distilled or bottled water (ca. 50 mL x 2) just prior to drinking it rapidly as a bolus, (b) encapsulated without excipient in clear gel-caps (50 μmol/dose), or (c) encapsulated in opaque green gel-caps as described above, by Xymogen (Orlando, FL). FDBS were encapsulated without excipients in clear gel-caps (either standard- or acid-resistant), or mixed, just prior to consumption, with 50% water, 46.5% pineapple juice (Dole): 3.5% lime juice (Safeway) [15,26]. BSdP without excipients was delivered in clear gel-caps.

Since our intent was to examine the effect of consumption of different broccoli preparations on urinary dithiocarbamate (DTC) excretion as a proxy for bioavailability, in most of the sub-studies reported herein we used only 5 subjects who could be available for an extended period of time (4 females and 1 male; mean age 53.8 yrs, range 32–62; 1 African-American, 4 white). The comparison of commercial supplements utilized 3 of these subjects plus another 17 that were recruited specifically for that study. All of the subjects were in good health, and had not recently taken antibiotics. Overall statistics were: 17 female/5 male; mean age about 51.3 yrs; 9 African-American, 13 white. At the time of each visit (sub-study or replicate), each subject was provided a detailed explanation of the study, and signed an informed consent form. The protocols for the studies reported herein (NA_00085573 and NA_00088662) were approved by the Institutional Review Board (IRB) of the Johns Hopkins University.

### Outcome Measurements

***Urinary DTC*** content of the pre-dose and 24 hr urines were measured using the cyclocondensation reaction [13]. Briefly, 100 and 200 μl of urine were incubated in 2.0 ml of 50% methanol, 50% water, 100 mM sodium borate, pH 9.2 and 16.3 mM 1,2-benzenedithiol in a sealed 4 ml vial for 2 hr at 65°C. After reaching room temperature and centrifugation, 200 μl from each vial was injected onto a 100 x 4 mm ODS Hypersil column (Thermo Scientific, Waltham, MA, USA) at 23°C and eluted with 80% methanol: 20% water at 0.5 ml/min for 10 min. The absorption peak area of the cyclocondensation product (1,3-benzodithiole-2-thione), was recorded at 360 nm, and the concentration in the urine samples was determined by comparison with the peak areas of authentic, recrystallized 1,3-benzodithiole-2-thione standards. The DTC contents of the pre-dose urines are expressed as nanomoles/mg of creatinine and that of the 24-hr urines as total μmol (**S1 Table**).

***Subject compliance*** with the protocol (adherence to the dietary restrictions and complete collection of 24-hr urines) was validated by direct querying of subjects upon delivery of 24-hr urine collections, and measurement of both urine volume and creatinines in the returned 24-hr urine collections and creatinines in the pre-dose urines. Creatinine measurements were performed by Hagerstown Medical Laboratory, (Hagerstown, MD, USA).

### Data Collection and Statistics

In all experiments, urine analyses were performed on coded samples, and investigators were blind to subject identity. In the comparison of commercial supplement with JHU-made extract, investigators were also blind to source of GR-rich supplement. Tests for normality and other statistics including determination of significant differences by repeated measures analysis of variance (anova), by oneway anova with Bonferroni adjustment and Bartlett’s test for equal variances, and by two-tailed t-tests with unequal variance, were performed using Stata ver. 11.2 (StataCorp, College Station, TX). Box and whisker plots identify mean, 25–50% range, 50–75% range (within box), upper and lower 95^th^ percentiles (whiskers) and actual data points are plotted.

## Results and Discussion

**Fig 1:**
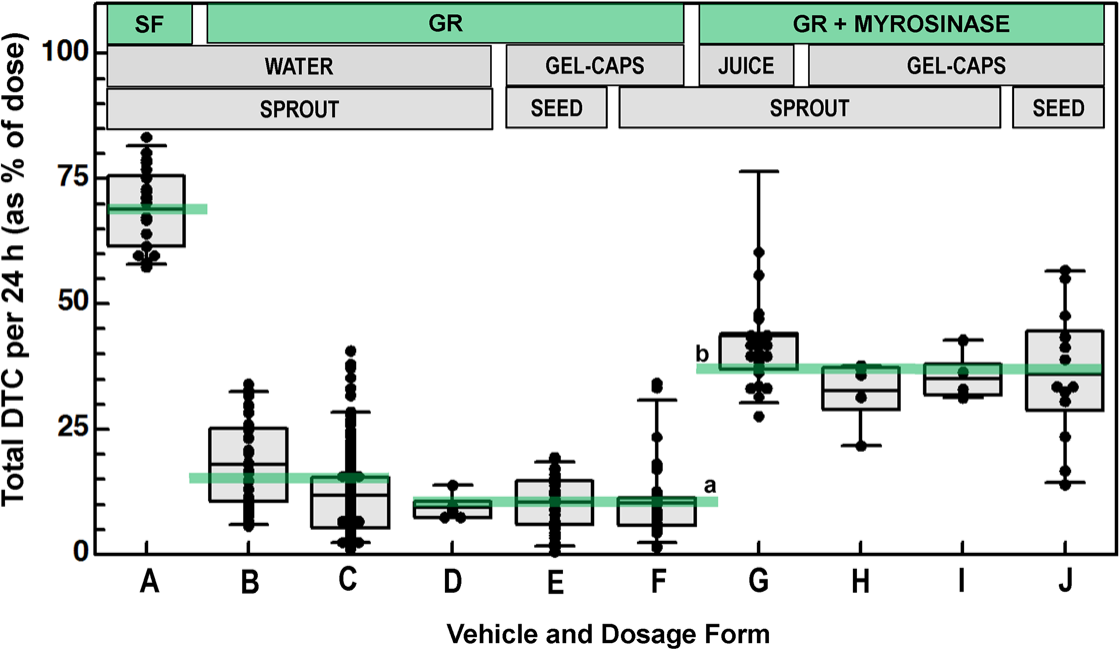
Mean daily excretion / conversion of SF- or GR-rich broccoli preparations to SF metabolites. Preparations are: SF-, GR-, or GR plus Myrosinase-rich; they are delivered either in water, juice, or gel-caps; and they are derived from either sprouts or seeds, as indicated at top of graph. Boxes around data points delineate the 25^th^ to 75^th^ percentile of values, and whiskers delineate the 5^th^ and 95^th^ percentiles. Solid line through middle of box is the mean. “Cohort numbers” in the following are from **Table 1**: **(A)** Hospitalized subjects given SF-rich BSE (mean 68.9%, data from Ref. [19]); **(B)** Hospitalized subjects given GR-rich BSE (mean 18.0%, data from Ref. [19]); **(C)** Free-living subjects given GR-rich BSE (mean 11.8%, data from Ref. [7]); **(D)** Cohort 1 (BSE in water; mean 9.4%); **(E)** Cohort 3 (Xymogen BSdE in gel-caps; mean 10.4%); **(F)** Cohort 2 (JHU BSE in gel-caps; mean 10.3%); **(G)** Cohort 6 (FDBS in dilute pineapple-lime juice; mean 44%); **(H)** Cohort 5 (FDBS in acid-resistant gel-caps; mean 32.7%); (**I)** Cohort 5 (FDBS in regular gel-caps; mean 35.1%); **(J)** Cohort 4 (BSdP; 35.9%). There is a highly significant difference between the means of D through F^a^ (10.3%), and G through J^b^ (39.6%), (p = 0.0000, by oneway anova).

The mean expected recovery of dithiocarbamates (sulforaphane (SF) and all of its metabolites) from 24 hour urine collections, following a single 8 AM dose of SF-rich broccoli extract (BSE) was about 69% of dose on a molar basis (data from previous study) [19], whereas recovery was only about 10% of dose following administration of a glucoraphanin (GR)-rich bolus delivered either in water or in gel-caps, derived from broccoli seeds or from sprouts, and from commercial supplements (OncoPLEX™, produced by Xymogen) or made by the investigators at Johns Hopkins University (**Fig 1**and **Table 1**). Bioavailability was variable, both between- and within- individuals as we have shown previously [7,19] (**S1 Fig**). Normal distribution was verified for each of the cohorts shown in **Table 1**.

**Table 1:** Mean bioavailability of sulforaphane from broccoli sprout and seed preparations rich in glucoraphanin, as affected by matrix, mode, of delivery, and activity of myrosinase on its substrate.

No.	Cohort	Matrix	Mode of Delivery	Dose (μmol GR)	Mean Availability (as % of dose)	Mean Availability (as % of SF delivered)	No. Subj. Enrolled	No. Subjects Assessed^f^
1	1	BSE^a^	dissolved in water	50	9.4	-	5	5
2	2	BSE	in gelcaps	69	12.8	-	20	16
3	2	BSE	in gelcaps	230	8.3	-	20	18
4	3	BSdE^b^	in gelcaps	69	11.2	-	20	17
5	3	BSdE^b^	in gelcaps	230	9.7	-	20	19
6	4	BSdP^c^	in standard gelcaps	100	32.6	-	5	4
7	4	BSdP	in standard gelcaps	100	31.7	-	5	4
8	4	BSdP	in standard gelcaps	100	44.1	-	5	4
9	5	FDBS^d^	in standard gelcaps	100	35.1	-	5	5
10	5	FDBS	in acid-resistant gelcaps	100	32.7	-	5	5
11	6	FDBS	pre-hydrolyzed^e^ in pineapple-lime juice	50	33.6	n.d.	5	5
12	6	FDBS	pre-hydrolyzed in pineapple-lime juice	50	48.4	97.6	5	5
13	6	FDBS	pre-hydrolyzed in pineapple-lime juice	50	40.2	91.4	5	4
14	6	FDBS	pre-hydrolyzed in pineapple-lime juice	100	41.8	84.3	5	4
15	6	FDBS	pre-hydrolyzed in pineapple-lime juice	200	40.9	83.9	5	5

^a^ Broccoli Sprout Extract

^b^ Broccoli Seed Extract produced commercially as OncoPLEX™ (from Xymogen)

^c^ Broccoli Seed Powder (with active myrosinase)

^d^ Freeze-Dried Broccoli Sprouts (with active myrosinase)

^e^ Myrosinase-converted for 10' at room temperature in juice

^f^ A subject pool of 5 volunteers participated in most tests described (numbers 1–15 above). They were augmented with another 17 volunteers for tests numbered 2–5 above.

Previously, we administered 200 μmol of GR-rich BSE to a large number of human subjects [7]. This preparation contained no active myrosinase, and therefore, hydrolysis of the GS presumably depended entirely upon action by the intestinal microflora. The 24-hr urine DTCs from these subjects ranged from 1 to 40% of the dose, but the mean was 11.5%. The results from a subset of these subjects who participated in ≥5 repeated administrations of GR is included as **S1 Fig**, and summary results are included in **Fig 1**for comparison (mean—18% of dose).

### GR-Rich BSE

In the present study, the same BSE was administered for the purpose of obtaining a baseline conversion efficiency on a new cohort of 5 subjects (**Table 1**, Cohort 1). The 5 subjects were each given a 50 μmol dose of GR-rich BSE, lacking active myrosinase (**S2 Table**), in 100 ml of water. The 24 hr urine DTC levels are presented in **Table 1**. Recoveries ranged from 3.7 to 6.9 μmol with a mean of 4.7 μmol (9.4% of dose).

### GR-Rich BSE vs. GR-Rich Supplements

**Fig 2:**
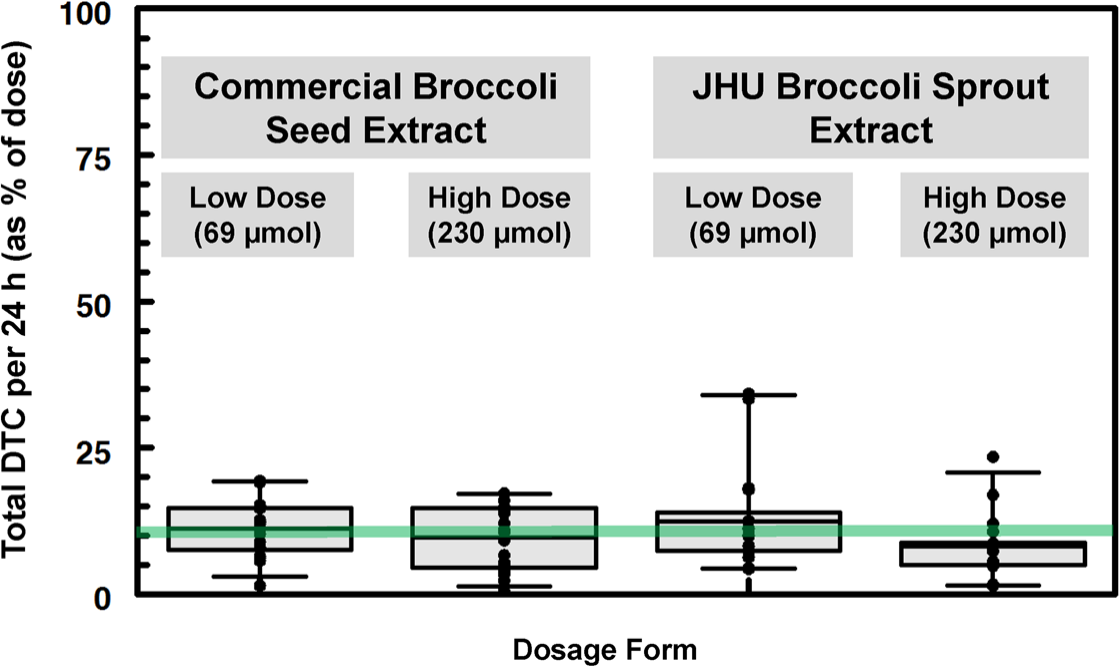
Mean daily excretion / conversion of GR-rich broccoli preparations to SF metabolites. Preparations are: Commercial broccoli seed extract (BSdE) formulated into gel-caps at 69 and 230 μmol/dose (10.4% conversion overall; **Table 1**, Cohort 3), and JHU BSE formulated (by Xymogen) into molar equivalent doses (10.3% conversion overall; **Table 1**, Cohort 2). There is no difference among the treatment groups overall (p = 0.0651 by repeated measures anova)

We also compared the bioavailability of BSE to that of a commercially prepared GR-rich supplement (e.g. a dry broccoli seed extract containing no active myrosinase; **S2 Table**) at two separate doses. Both GR-sources were formulated into opaque green gel-caps using excipients standard to the supplement industry, so that they could be singulated into capsules by machine. These sources were equally bioavailable (10.4 and 10.3% respectively; p = 0.2340), and in each case the higher dose was nominally, but not significantly less available than the lower dose (9.7% and 11.2%, p = 0.412; 8.2% and 12.5%, p = 0.0956, for differences between high and low doses of the commercial supplement and the JHU powder, respectively) (**Fig 2**).

### Freeze-Dried Broccoli Sprouts (FDBS) with Active Myrosinase

Five consecutive challenges were then conducted in which the same subjects were given freeze-dried broccoli sprouts (FDBS) in juice in which the glucosinolates had been hydrolyzed to isothiocyanates by the endogenous myrosinase prior to ingestion. The first 3 challenges contained 50 μmol of GR, then 100 μmol, and finally 200 μmol. For the 50 μmol doses, urinary DTCs ranged from 13.8 to 30.1 μmol (mean—20.5 μmol or 41.0% of dose). This recovery is over 4-fold higher than the mean DTC recovery following 50 μmol BSE doses. For the 100 μmol dose, recovery ranged from 40.3 to 43.3 μmol (mean—41.8 μmol or 41.8% of dose), and for the 200 μmol dose, DTC recovery ranged from 66.3 to 111.5 μmol (mean—81.7 μmol or 40.8% of dose). These results confirm that there is a linear relationship between GR dose and DTC content of the urine, as has been reported previously [7,14]. The SF content of these doses which were allowed to autolyze for 10 min after mixing with juice, were measured by cyclocondensation of small aliquots removed just prior to ingestion (**Table 1**). For the 50, 100 and 200 μmol GR doses, the corresponding mean DTC levels were 23.5, 49.6 and 97.4 μmol, indicating that *in vitro*, only about 50% of the starting GR was hydrolyzed to SF. Once the dose was ingested, 86% of the isothiocyanates were recovered in the urine as DTC, in close agreement to previously published work on bioavailability of SF [1,13,14,19,20].

We then sought to determine whether bioavailability would be the same if this preparation was not pre-treated with myrosinase, but rather, the myrosinase was permitted to act post-ingestion (in the gastrointestinal tract). FDBS (100 μmol) were packed into either regular clear gelatin capsules (gel-caps), or acid-resistant capsules designed not to release their contents until passage from the stomach. With standard capsules 24-hr urine DTC ranged from 31.3 to 42.8 μmol (mean—35.1% of dose), and with acid resistant capsules the range was 21.7 to 37.7 μmol (mean—32.7% of dose). These results show that significant hydrolysis of the glucosinolates by the endogenous myrosinase did occur in the gastrointestinal tract but also suggest that whether dissolution of the capsule occurred in the stomach or further along in the gastrointestinal tract made little difference in the extent of hydrolysis. When compared with the 100 μmol pre-hydrolyzed FDBS dose in juice (41.8% of dose), these 24 hr urine DTC recoveries were only slightly lower.

**Fig 3:**
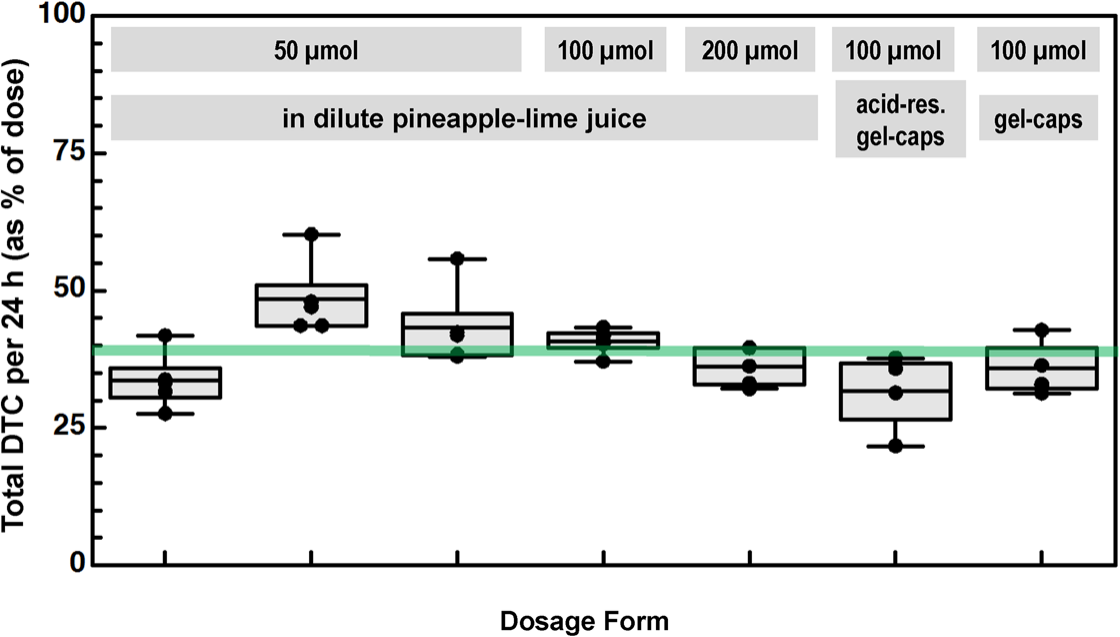
Mean daily excretion / conversion of freeze-dried broccoli sprouts (FDBS) containing active myrosinase, to SF metabolites. (see also Table 1, Cohort 4). Preparations were either pre-mixed in dilute pineapple-lime juice for 15 min at room temperature to allow autolysis in-vitro, and then consumed directly by volunteers (40.5% conversion), or swallowed in either acid-resistant or standard gel-caps (33.8% conversion). The same 5 subjects performed each test. There was no effect of subject on conversion (p = 0.2740) by repeated measures analysis of variance. There was a significant difference (by oneway anova with Bonferroni adjustment and Bartlett’s test for equal variances), between replicates at the 50 μmol dose level (p = 0.0107), but no significant difference between dose levels (pooled replicates; p = 0.3329) or dose matrices (p = 0.3735 between capsule types and p = 0.0255 between capsules and juice).

**Fig 4:**
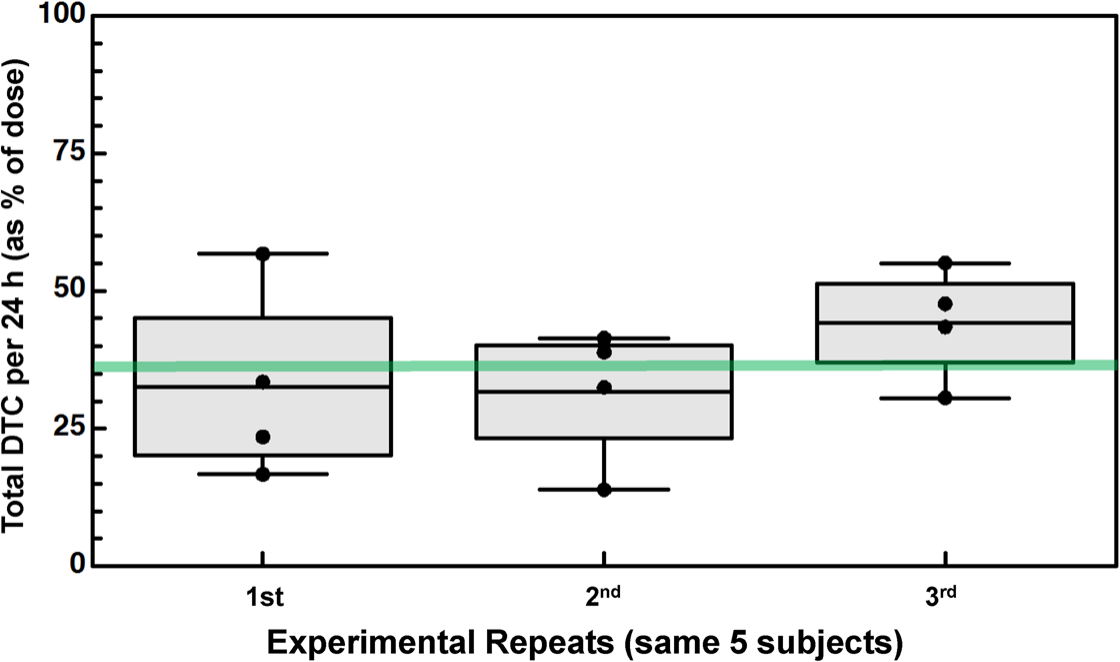
Mean daily excretion / conversion of broccoli seed powder (BSdP) containing active myrosinase, to SF metabolites. Preparations were delivered in standard gel-caps. The same 5 subjects performed each test (**Table 1**, Cohort 4). There was no significant difference between replications (p = 0.3747 using a repeated measures analysis of variance). The mean conversion of all 3 replicates was 36% of dose.

In contrast to the availability from SF- or GR-rich extracts, when an active source of the enzyme myrosinase (which converts GR to SF both in the plant, *in vitro*, and *in vivo*) was added in the form of either broccoli sprouts or seeds, about 40% of the dose was recovered following delivery either in juice, standard gel-caps, or acid-resistant gel-caps (**Table 1**and **Figs 1, 3**and **4**). Freeze-dried broccoli sprouts alone contains substantial endogenous myrosinase activity (**S2 Table**) that is sufficient to convert GR to SF either when mixed in a dilute pineapple-lime juice carrier [15] just prior to dosing, when packaged in gel-caps that are presumed to release their contents to the stomach, or when packaged in acid-resistant gel-caps calibrated to release their contents only upon passage to the upper small intestine (**Fig 3**). Bioavailability in juice (40%) was about the same as bioavailability in capsules (34%).

### Broccoli Seed Powder (BSdP) with Active Myrosinase

Having established that delivery in capsules provided similar bioavailability to pre-mixing with juice (or water), we evaluated the bioavailability of broccoli seed powder (also containing substantial endogenous myrosinase activity sufficient to convert the entire GR titer to SF), delivered in gel-caps. Mean conversion to DTC urinary metabolites was 36.1%, based upon 3 temporally spaced administrations in which 5 persons were administered a single 100 μmol dose of ground broccoli seed powder and conversion efficiency was 32.6, 31.7, and 44.1% respectively (**Fig 4**). Whereas stability of the other preparations has been documented previously [6,7,14–16,19,20,27–29] the stability of myrosinase in ground seeds has not heretofore been carefully examined. We have verified that after a year in storage at household freezer, refrigerator, or room temperature conditions, it remains fully active (**S2 Fig** and **S2 Table**), and GR titer remains unchanged also (**S3 Fig**).

There was perfect compliance with the dosing regimen, because volunteers were provided their single bolus of broccoli-based GR by the investigators, who watched them consume it. Compliance of free-living subjects with dietary proscriptions was more difficult to ascertain, but assessment of DTC pre-dose and measurement of urine volumes gave us good confidence of compliance in most collections (**S1 Table)**. Of the 135 planned assessments in **Table 1**, 15 of them (11%) were ultimately censored due to apparent lack of rigorous compliance with study procedures. Less easily ascertained are subjects’ actions involving environmental influences that may or may not have been proscribed (e.g. antibiotic use is forbidden, but other habits such as smoking or frequency of exercise or alcohol consumption, that affect the gut microbiome or otherwise impact GR-to-SF conversion and subsequent metabolism may have been overlooked).

For many years we have produced, tested, validated, formulated, singulated, and supplied broccoli sprout extract powder for our use, and that of our collaborators, in clinical studies [6,7,10,15,19,27–31]. We have now discontinued making BSE, because there are several high quality, commercially available broccoli supplements on the market, whereas this was not the case when our early clinical work with broccoli sprouts commenced. We have tested and found some to be of high quality, however, others are of poor quality. There are increasing numbers of participants in clinical studies who demand continued access to supplements after the studies in which they were enrolled have ended. In contrast to something made by Johns Hopkins scientists which is unsustainable in the long term, the development of clinical information on a commercial product, (typically not provided by the manufacturers), provides an opportunity to expand the menu of high quality supplements not only for our participants/volunteers, but for future investigators, and for the general public who look to credible institutions (both regulatory and academic) to develop standards of product safety and bioavailability.

In conclusion, we have examined several broccoli preparations and delivery vehicles for their suitability for use in clinical trials, and their potential to deliver SF to persons whom may ultimately wish to consume broccoli-based supplements. Pre-hydrolysed FDBS in which GR was delivered after conversion to SF in juice containing vitamin C and able to partially mask the flavor of broccoli, provided the highest bioavailability on a molar basis (about 40%). FDBS or BSdP in capsules in which the dose was administered as GR and was dependent on hydrolysis by the plant myrosinase, but modulated by subject gastrointestinal flora, was only slightly less bioavailable by the same metric (about 35%). On the other hand, a BSE containing GR without plant myrosinase showed substantially lower conversion to SF (about 10%), consistent with previously published data. Thus, whereas matrix effects were minimal, the presence of active myrosinase led to substantial and significant enhancement of sulforaphane bioavailability.

## Supporting Information

S1 Fig Mean daily excretion / conversion of BSE prepared by JHU, to SF metabolites (200 μmol/dose). Only subjects who repeated the test 5 or more times in our previously published study [7] are included here in order to illustrate the low, and variable conversion anticipated when GR is delivered orally.
(TIFF)

S2 Fig Stability of broccoli seed powder: myrosinase activity. (TIFF)

S3 Fig Stability of broccoli seed powder: glucoraphanin titer. (TIFF)

S1 Table Pre- and post-dose DTC and creatinine values, and urine volumes for subjects enrolled in Cohorts 1–6 of Table 1. (XLSX)

S2 Table Myrosinase activity measured in representative samples of the four glucoraphanin-rich preparations utilized in this study. (DOCX)

## Abbreviations

ARE: antioxidant responsive element
BSE: broccoli sprout extract
BSdE: broccoli seed extract
BSdP: broccoli seed powder
DTC: dithiocarbamates
FDBS: freeze-dried broccoli sprouts
GMP: good manufacturing practice(s)
GR: glucoraphanin
JHU: Johns Hopkins University School of Medicine
Keap1: Kelch-like ECH-associated protein 1
Nrf2: nuclear factor erythroid 2 p45-related factor 2
SF: sulforaphane
