# Supplementary figures and images for: Sulforaphane Bioavailability from Glucoraphanin-Rich Broccoli: Control by Active Endogenous Myrosinase
Source: PLoS One. 2015 Nov 2;10(11):e0140963. doi: 10.1371/journal.pone.0140963 (PMC4629881; doi:10.1371/journal.pone.0140963)

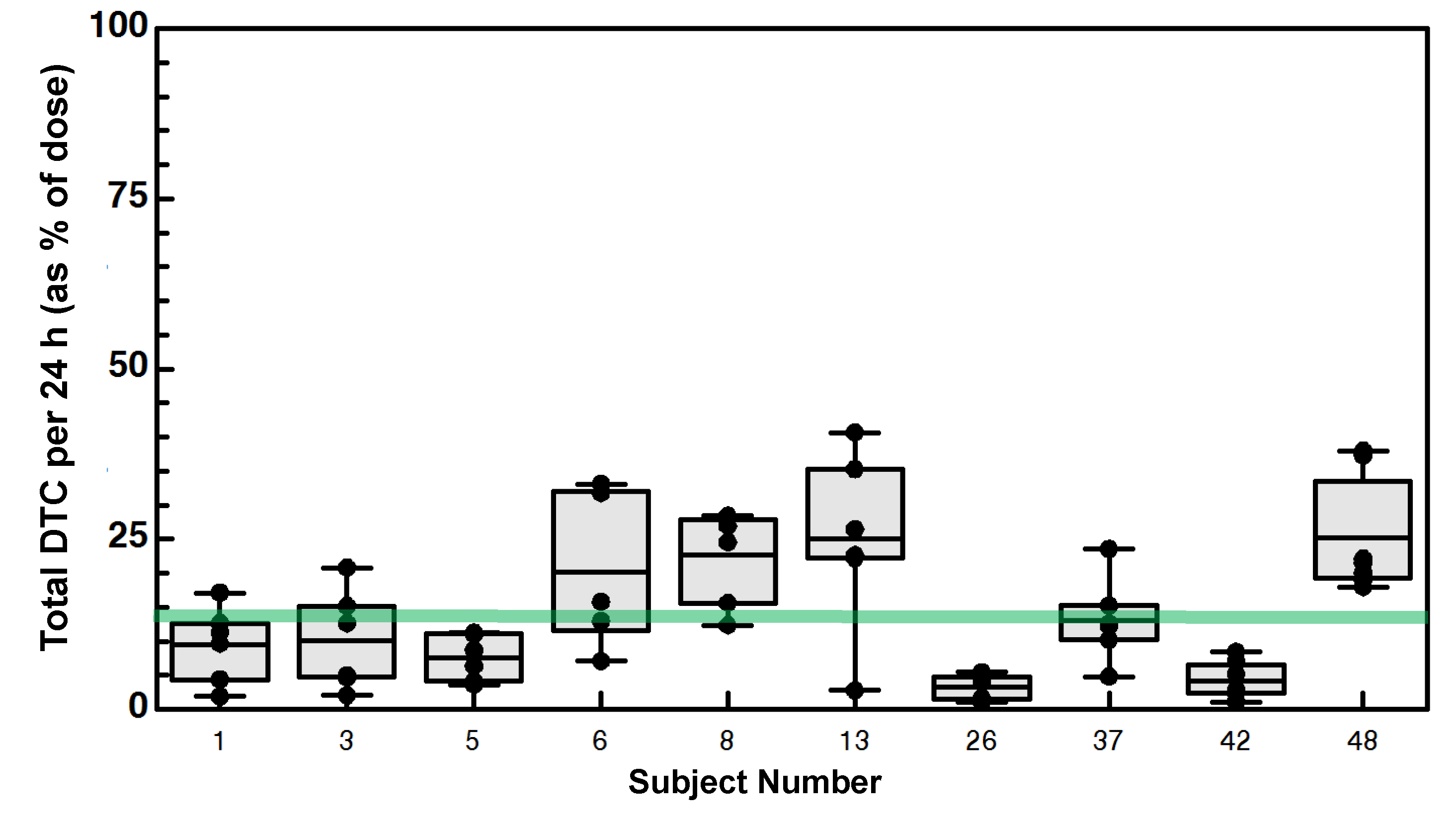

Supplement: S1 Fig — Only subjects who repeated the test 5 or more times in our previously published study [7] are included here in order to illustrate the low, and variable conversion anticipated when GR is delivered orally. (TIFF) [file pone.0140963.s001.tiff]

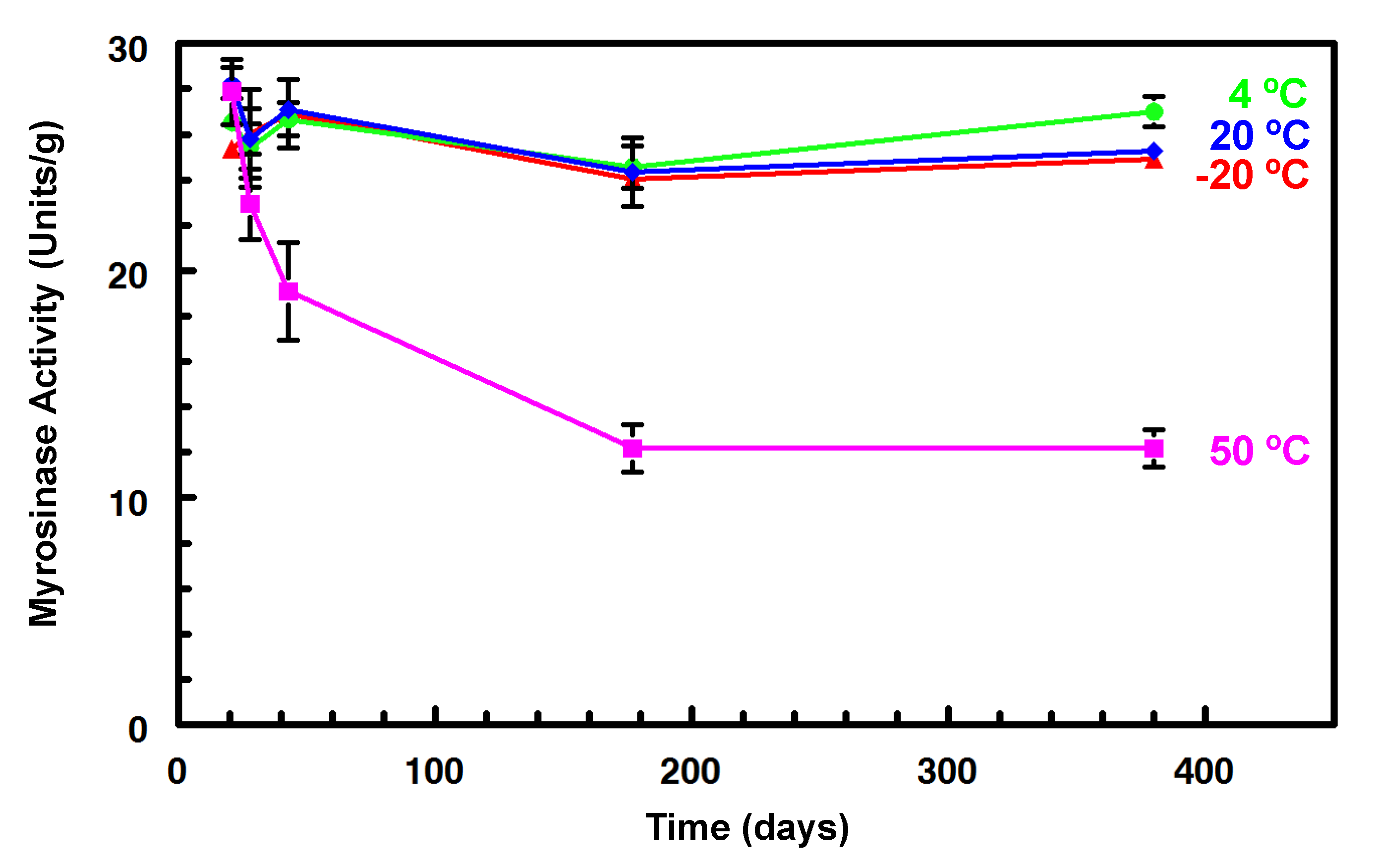

Supplement: S2 Fig — (TIFF) [file pone.0140963.s002.tiff]

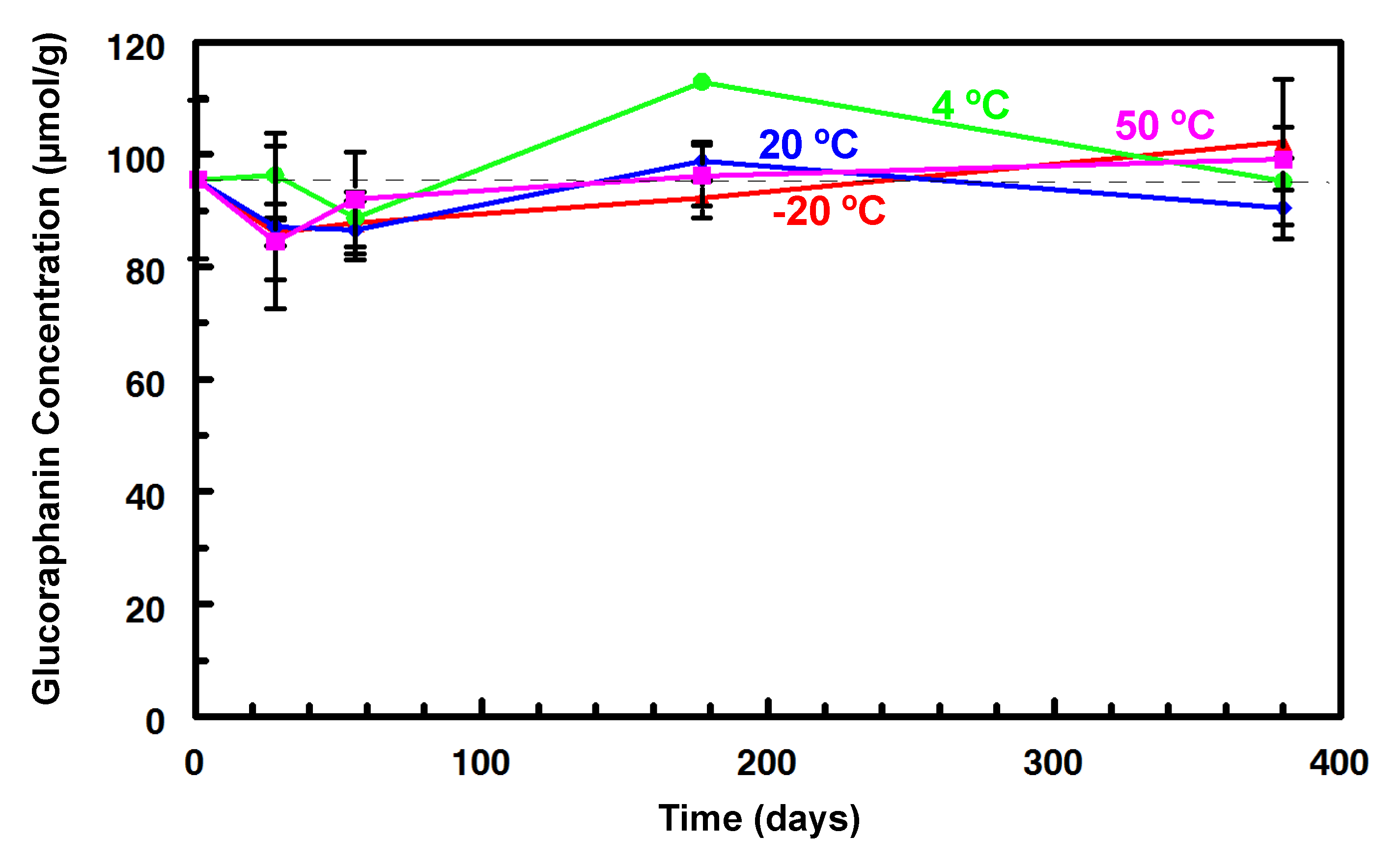

Supplement: S3 Fig — (TIFF) [file pone.0140963.s003.tiff]
