# Supplementary material for: Sulforaphane Bioavailability from Glucoraphanin-Rich Broccoli: Control by Active Endogenous Myrosinase
Source: PLoS One. 2015 Nov 2;10(11):e0140963. doi: 10.1371/journal.pone.0140963 (PMC4629881; doi:10.1371/journal.pone.0140963)
Supplement: S2 Table — (DOCX) [file pone.0140963.s005.docx]

**S2 Table**

**S2 Table.** Myrosinase activity measured in representative samples of the four glucoraphanin-rich preparations utilized in this study.

**Myrosinase Activity**

**Glucoraphanin (GR) Matrix (Units/gram ± 1 S.D.)**

GR-rich broccoli sprout extract (BSE) 0

GR-rich commercial supplement 0

Freeze-dried broccoli sprouts (FDBS) 103 ± 0.66

Broccoli seed powder (BSdP) 28.1 ± 0.67
